# Trends of primary liver cancer incidence and mortality in the United States: A population-based study over the last four decades

**DOI:** 10.1371/journal.pone.0309465

**Published:** 2024-09-05

**Authors:** Saryia Adra, Yousef Alabrach, Anas Hashem, Amir Mahmoud, Amani Khalouf, Ahmed El-khapery, Ali Abdelhay, Mohamad Mansour, Batool Aldaher, Hiba Barqawi, Eman Abu-Gharbieh

**Affiliations:** 1 College of Medicine, University of Sharjah, Sharjah, United Arab Emirates; 2 Sheikh Khalifa Medical City, Abu Dhabi, United Arab Emirates; 3 Rochester General Hospital, Rochester, New York, United States of America; 4 Tawam Hospital, Abu Dhabi, United Arab Emirates; 5 Research Institute of Medical and Health Sciences, University of Sharjah, Sharjah, United Arab Emirates; Osaka Rosai Hospital: Osaka Rosai Byoin, JAPAN

## Abstract

**Background:**

Primary liver cancer is the third leading cause of cancer deaths worldwide and has one of the worst 5-year survival rates. This study examines US primary liver cancer incidence and incidence-based mortality trends over four decades.

**Research design and methods:**

The SEER-9 registry was used to study primary liver cancer cases from 1978 to 2018. The incidence and mortality rates were calculated based on gender, age, race, and stage of diagnosis. Joinpoint regression software was used to calculate the annual percent change.

**Results:**

The overall incidence rate of primary liver cancer from 1978 to 2018 increased by 2.71%/year (p<0.001). Rates in patients <50 years old began to fall in 2002 at a rate of -3.62%/year (p<0.001). Similarly, the incidence-based mortality rates for primary liver cancer increased by 2.15%/year (p<0.001). Whereas Whites incidence-based mortality rates began to plateau in 2012 (0.18%/year; p = 0.84), Blacks rates have declined since 2010 (-2.93%/year; p = 0.03), and Asian rates have declined since 1999 (-1.30%/year; p<0.001).

**Conclusion:**

While the overall primary liver cancer incidence and incidence-based mortality have been increasing over the last four decades, there was an observed decline in incidence and incidence-based mortality in recent years, especially among at-risk subgroups.

## Introduction

Primary liver cancer (PLC) is the seventh most diagnosed cancer and the third leading cause of cancer-related death worldwide, causing an estimated 830,180 deaths in 2020 [1]. Worldwide, the PLC incidence rates continue to rise, particularly in the United States (US). Additionally, the 5-year survival rates remain one of the worst among all cancers, with rates below twenty percent [2, 3].

The two main subtypes of PLC are hepatocellular carcinoma (HCC) and intrahepatic cholangiocarcinoma (ICC), with HCC comprising around eighty-five percent of all liver cancer cases [4]. HCC primarily complicates longstanding cirrhosis, exacerbated by factors such as chronic viral hepatitis, alcoholic and non-alcoholic fatty liver disease, as well as aflatoxin exposure [5]. While risk factors for ICC are less specific, they also include cirrhosis, viral hepatitis, primary sclerosing cholangitis, and liver fluke infections (in Asia) [6]. Regional and age-related variations exist for different exposures and are reflected in the changing incidence trends observed within these subgroups [7]. Implementation of early screening protocols, large public health initiatives, and advanced management protocols for the underlying liver disease has significantly impacted the incidence and survival rates of HCC [8]. This has translated into early detection and improved survival of HCC patients in the last two decades [9]. Reports on the decreasing rates seen among certain at-risk subgroups of HCC suggest that there could be a decline in the incidence of PLC in the near future [10].

The Surveillance, Epidemiology, and End Results (SEER) program of the National Cancer Institute has been an essential resource for cancer epidemiology for decades [11]. This continuous monitoring of disease patterns and population characteristics has allowed for an increased understanding of the burden of PLC, better assessment of the quality of healthcare delivery, and identifying gaps in preventive measures. Quantitative analysis of incidence rates and etiology-based survival in HCC patients has been reported [5, 12]. PLC incidence-based rates (IBR) and incidence-based mortality rates (IBMR) were examined over the last four decades using the SEER database. Henceforth, this article aims to add an updated understanding of the changing trends of PLC in the US.

## Material and methods

The incidence and mortality data for primary liver cancer (PLC) were obtained from SEER-9, a population-based cancer registry that covers approximately 9.4% of the US population from 1978 to 2018 [11]. Cases were identified using specific International Classification of Diseases for Oncology, 3^rd^ edition, codes (site: C22.0 and C22.1; histology: 8160, 8162, 8170–8175, and 8180). Only cases of malignant PLC were included in the analysis, while cases without active follow-up or those diagnosed solely based on autopsy or death certificates were excluded.

Staging information was consolidated using a merged variable. From 1973 to 2015, the ’SEER historic stage A’ was utilized, while from 2016 to 2018, the ’Combined summary stage (+2004)’ was employed.

### Statistical analysis

Both incidence-based mortality rates (IBMR) and incidence-based rates (IBR) were age-standardized to the 2000 US population. The calculation of IBMR and IBR took into account variables such as age, sex, ethnicity/race, and stage at the time of diagnosis. IBMR was determined by dividing the number of PLC deaths among cases by the person-time at risk within the SEER sites [11]. Population adjustments for the Katrina and Rita Hurricanes were not applied.

To examine trends over time, annual percent changes (APCs) were estimated using Joinpoint regression. This statistical analysis identifies the calendar years with significant annual percentage changes by selecting the optimal log-linear regression model with the minimum number of joinpoints required to accurately fit the data [13]. The model with the fewest number of Joinpoints, yet providing the best fit, was selected for analysis [14].

### Ethics approval and consent to participate

This study is based on the use of de-identified public data from the SEER database. It does not involve interaction with human subjects or the use of personal identifying information. The study did not require informed consent from the SEER-registered cases, and the authors obtained Limited-Use Data Agreements from SEER. No trial registration was necessary.

## Results

### PLC incidence-based rates

**Fig 1:**
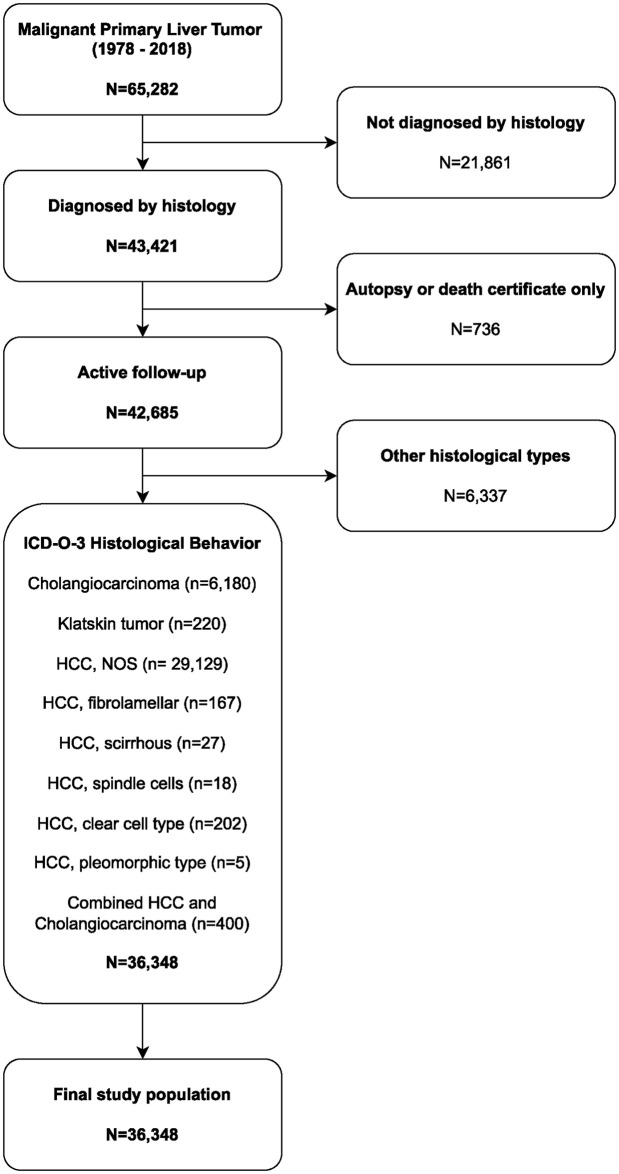
Study flowchart design and patient selection.

Malignant PLC was diagnosed in 65,282 patients between 1978 and 2018. Our study included 36,348 of them (Fig 1). The majority of the cases (71.39% [n = 25,949]) were men, 66.44% [n = 24,148] were White, 89.61% [n = 32,573] were over 50, and 34.22% [n = 12,439] had localized cancer. The liver (85.25% [n = 30,985]) was the most common subsite for PLC, followed by the intrahepatic biliary duct (14.75% [n = 5,363]). Table 1 summarizes the patient demographics and age-standardized IBR.

**Table 1:** Age-standardized incidence rates and annual percent changes in primary liver cancer rates, 1978–2018.

Incidence-based Rates													
		Segment 1		Segment 2		Segment 3		Segment 4		Segment 5		Incidence rates Per 100,000	
	AAPC (95% CI)	Years	APC (95% CI)	Years	APC (95% CI)	Years	APC (95% CI)	Years	APC (95% CI)	Years	APC (95% CI)	2017	2018
**Overall**	2.71 (2.40, 3.20)	1978–2007	3.86 (3.54, 4.18)	2007–2018	-0.26 (-1.07, 0.56)							4.60	4.01**
**Sex**													
**Men**	2.52 (2.16, 2.89)	1978–2007	3.78 (3.41, 4.15)	2007–2018	-0.72 (-1.64, 0.21)							6.96	6.11**
**Women**	2.73 (2.32, 3.13)	1978–2008	3.43 (3.06, 3.81)	2008–2018	0.64 (-0.57, 1.86)							2.56	2.21**
**Age, years**													
**<50**	1.13 (0.48, 1.78)	1978–2002	4.41 (3.58, 5.25)	2002–2018	-3.62(-4.71, -2.51)							0.44	0.46
**≥50**	2.91 (2.58, 3.25)	1978–2008	3.91 (3.59, 4.24)	2008–2018	-0.03(-1.00, 0.95)							15.48	13.33**
**Race/ethnicity**													
**White**	2.80 (1.31, 4.31)	1978–1986	1.52 (-1.44, 4.58)	1986–1998	5.75 (4.29, 7.22)	1998–2001	-1.50 (-16.55, 16.27)	2001–2008	4.77 (2.13, 7.49)	2008–2018	0.33 (-0.67, 1.34)	4.09	3.55*
**Black**	2.14 (1.55, 2.73)	1978–2008	3.47 (2.89, 4.04)	2008–2018	-1.75 (-3.42, -0.06)							5.94	5.23
**Other races**	0.28 (-0.12, 0.68)	1978–2005	1.80 (1.34, 2.26)	2005–2018	-2.80 (-3.61, -1.98)							6.42	5.37**
**Stage**													
**Localized**	3.95 (2.97, 4.94)	1978–1988	2.59 (-0.33, 5.60)	1988–2006	8.40 (7.56, 9.24)	2006–2015	0.35 (-1.07, 1.79)	2015–2018	-6.13 (-11.96, 0.09)			1.95	1.76
**Regional**	2.91 (2.41, 3.41)	1978–2005	4.59 (4.01, 5.17)	2005–2018	-0.49 (-1.50, 0.53)							1.24	1.00**
**Distal** ***	1.98 (1.76, 2.21)											1.16	1.01

* Indicates statistical significance (p<0.05)

** Indicates significant declines from 2017 to 2018, p<0.05;

*** Model has no joinpoints; it only has a continuous trend.

AAPC = average annual percent changes, APC = annual percent changes, CI = confidence interval;

Rates were standardized to the 2000 US population in 5-year age categories.

The Joinpoint regression model requires a minimum of two observations between a joinpoint and the first or last data point and between two joinpoints.

**Fig 2:**
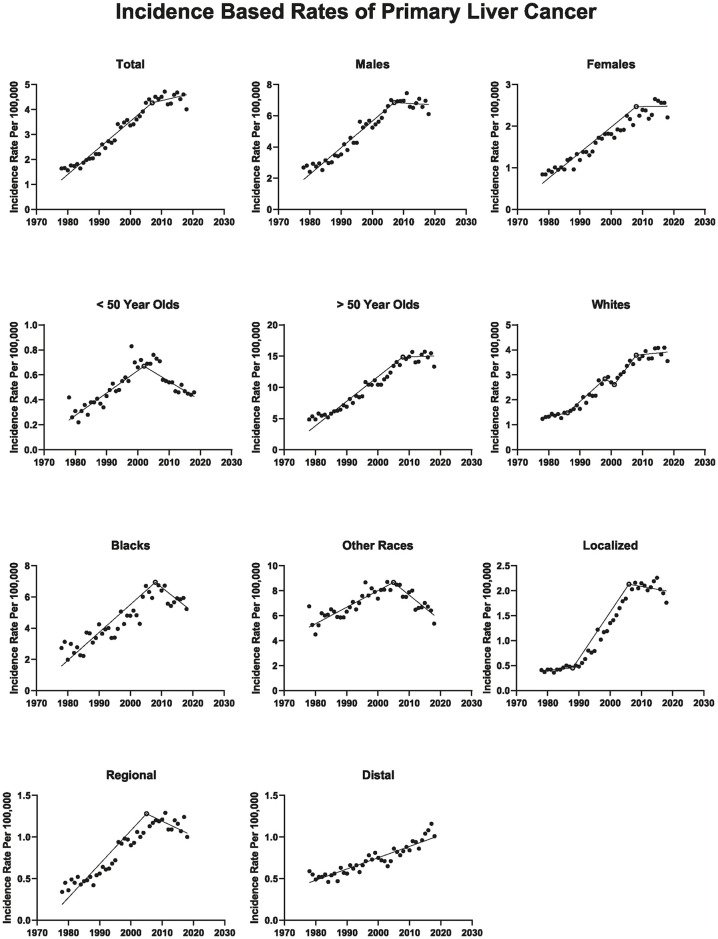
Age-standardized incidence rates of primary liver cancer in the SEER-9 US cancer registry, 1978–2018. Solid circles represent incidence rates, open circles represent years with a significant change in annual percent changes in rates, and lines represent modeled rates using Joinpoint regression. The y-axis varies between figures.

Overall, PLC rates increased by 3.86%/year (p < 0.001) during 1978–2007, then plateaued starting in 2007 (APC, -0.26%/year; p = 0.524). The average APC from 1978 to 2018 was 2.711% yearly (p < 0.001). Males’ and females’ rates increased up to 2007 and 2008, respectively, then plateaued (APC, -0.72%/year; p = 0.126; APC, 0.64%/year; p = 0.291, respectively). For ≥ 50-year-olds, rates increased during 1978–2008 (APC, 3.91%/year; p < 0.001), then plateaued from 2008 to 2018 (APC, -0.03%/year; p = 0.953). In contrast, for < 50-year-olds, rates increased during 1978–2002 by 3.91%/year (p < 0.001), then decreased from 2002 to 2018 (APC, -3.62%/year; p < 0.001) (Fig 2).

After years of steadily increasing PLC rates, it plateaued among white individuals beginning in 2008 (APC, 0.33%/year; p = 0.505) and declined among black individuals in 2008 (APC, -1.75%/year; p = 0.043). Starting in 2005, among the other races (American Indian/AK Native and Asian/Pacific Islander), PLC rates decreased by -2.80%/year (p < 0.001).

When looking at the IBR of localized PLC there was a significant increase between 1988 to 2006 (APC, 8.40%/year; p < 0.001), then plateaued (2006–2015) before decreasing from 2015 to 2018 (APC, -6.13%/year; p = 0.053). As for regional PLC IBR increased from 1978 to 2005 (APC, 4.60%/year; p < 0.001), then plateaued till 2018. Finally, distant PLC rates have been increasing throughout the study period (APC, 1.98%/year; p < 0.001).

When comparing the PLC IBR between 2017 to those in 2018, we found a significant decrease (RR, 0.87; 95% CI = 0.809–0.932; p < 0.001). Similarly, between 2017 and 2018, in analyses restricted to SEER-21 (RR, 0.96; 95% CI = 0.925–0.991; p = 0.01), SEER-18 (RR, 0.96; 95% CI = 0.922–1.000; p = 0.05) and SEER-13 (RR, 0.86; 95% CI = 0.818–0.921; p < 0.001) registries and when liver/intrahepatic bile duct rates were estimated using delay adjustment (RR, 0.93; p = 0.005).

### PLC incidence-based mortality rates

**Fig 3:**
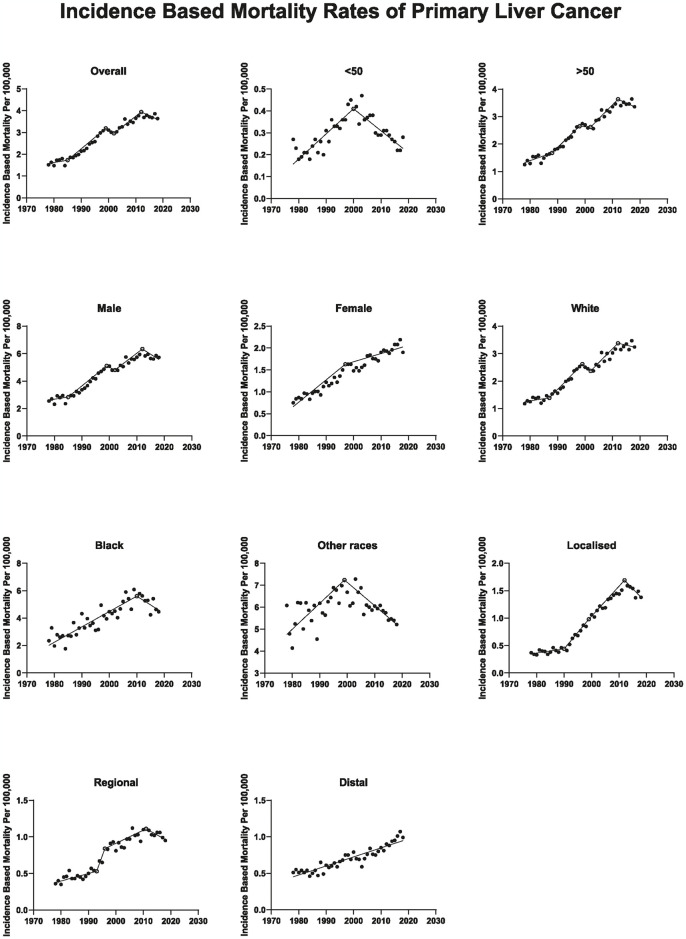
Age-standardized mortality rates for primary liver cancer in the SEER-9 US cancer registry, 1978–2018. Solid circles represent incidence rates, open circles represent years with a significant change in annual percent change in rates, and lines represent modeled rates using Joinpoint regression. The y-axis varies across figures.

The PLC IBMR increased by 1.11%/year (p = 0.321) during 1978–1985, then increased further to 4.56%/year (p < 0.001) between 1985 to 1999, followed by a slight decrease of -1.62%/year (p = 0.745) between 1999 and 2002, while from 2002 to 2012 the mortality increased to 2.41%/year (p < 0.001) then plateaued starting in 2012 (APC, -0.70%/year; p = 0.299). The average APC from 1978 to 2018 was 2.15%/year (p < 0.001). Males’ IBMR steadily increased up to 2012 and then plateaued from 2012 to 2018 (APC, -1.25%/year; p = 0.092). Whereas females’ rates increased from 1978 to 1997 (APC, 3.624%/year; p < 0.001), then increased at a slower rate to 2018 (APC, 1.651%/year; p < 0.001). For ≥ 50-year-olds, rates steadily increased from 1978 to 2012, then plateaued from 2012 to 2018 (APC, -0.52%/year; p = 0.490). In contrast, < 50-year-old rates increased during 1978–2000 by 3.94%/year (p < 0.001), then decreased from 2000 to 2018 (APC, -3.168%/year; p < 0.001) (Fig 3). Table 2 summarizes the patient demographics and age-standardized IBMR.

**Table 2:** Age-standardized incidence-based mortality rates, and annual percent changes in primary liver cancer rates, 1978–2018.

Incidence-based Mortality Rates													
		Segment 1		Segment 2		Segment 3		Segment 4		Segment 5		Incidence rates Per 100,000	
	AAPC (95% CI)	Years	APC (95% CI)	Years	APC (95% CI)	Years	APC (95% CI)	Years	APC (95% CI)	Years	APC (95% CI)	2017	2018
**Overall**	2.15* (1.24, 3.06)	1978–1985	1.11 (-1.14, 3.42)	1985–1999	4.56* (3.87, 5.25)	1999–2002	-1.62 (-11.15, 8.94)	2002–2012	2.41* (1.56, 3.27)	2012–2018	-0.70 (-2.03, 0.66)	3.86	3.64
**Sex**													
**Men**	1.94* (0.93, 2.95)	1978–1985	0.69 (-1.84, 3.28)	1985–1999	4.55* (3.78, 5.32)	1999–2002	-1.91 (-12.32, 9.74)	2002–2012	2.34* (1.40, 3.28)	2012–2018	-1.25 (-2.71, 0.22)	5.86	5.73
**Women**	2.58* (2.23, 2.94)	1978–1997	3.62* (2.97, 4.29)	1997–2018	1.65* (1.29, 2.01)							2.19	1.90
**Age, years**													
**<50**	0.68* (0.02, 1.34)	1978–2000	3.94* (2.99, 4.90)	2000–2018	-3.17* (-4.14, -2.19)							0.22	0.28
**≥50**	2.41* (1.59, 3.25)	1978–1988	2.23* (0.74, 3.74)	1988–1998	4.97* (3.61, 6.36)	1998–2002	-0.43 (-6.28, 5.79)	2002–2012	3.01* (2.02, 4.02)	2012–2018	-0.52 (-2.03, 1.01)	3.64	3.36
**Race/ethnicity**													
**White**	2.46* (1.26, 3.68)	1978–1987	1.42 (-0.56, 3.44)	1987–1999	5.37* (4.21, 6.54)	1999–2002	-3.37 (-15.85, 10.94)	2002–2012	3.17* (1.99, 4.35)	2012–2018	0.18 (-1.62, 2.01)	3.47	3.24
**Black**	1.63* (0.95, 2.32)	1978–2010	2.81* (2.25, 3.37)	2010–2018	-2.93* (-5.54, -0.23)							4.63	4.46
**Other races**	0.13 (-0.29, 0.55)	1978–1999	1.44* (0.74, 2.13)	1999–2018	-1.30* (-1.79, -0.80)							5.40	5.21
**Stage**													
**Localized**	3.50* (2.73, 4.28)	1978–1990	1.92* (0.26, 3.61)	1990–1999	9.44* (7.10, 11.83)	1999–2012	3.84* (3.06, 4.63)	2012–2018	-2.53* (-4.25, -0.77)			1.49	1.38
**Regional**	2.34* (0.55, 4.16)	1978–1993	2.23* (0.94, 3.53)	1993–1996	14.69 (-8.70, 44.08)	1996–2011	1.94* (1.10, 2.79)	2011–2018	-1.51 (-3.49, 0.51)			0.99	0.95
**Distal** **	1.88* (1.66, 2.10)											1.07	0.99

* Indicates statistical significance (p<0.05)

** Model has no joinpoints; it only has a continuous trend.

AAPC = average annual percent changes, APC = annual percent changes, CI = confidence interval;

Rates were standardized to the 2000 US population in 5-year age categories.

The Joinpoint regression model requires a minimum of two observations between a joinpoint and the first or last data point and between two joinpoints.

After years of steadily increasing PLC IBMR, it plateaued among white individuals beginning in 2012 (APC, 0.18%/year; p = 0.843) and declined among Black individuals in 2010 (APC, -2.925%/year; p = 0.034). Starting in 1999, among the other races (American Indian/AK Native and Asian/Pacific Islander), PLC rates decreased by -1.30%/year (p < 0.001).

The analysis of localized PLC IBMR showed an increase from 1978 to 1990, followed by a more rapid increase till 1999 that subsequently slowed till 2012 (APC, 1.92%/year; p = 0.025; APC, 9.44%/year; p < 0.001; APC, 3.84%/year; p < 0.001; respectively), then decreased from 2012 to 2018 (APC, -2.53%/year; p = 0.006). Regional PLC IBMR started to decline from 2011 to 2018 (APC, 1.51%/year; p = 0.136). Finally, distant PLC rates have been increasing throughout the study period (APC, 1.88%/year; p < 0.001).

### Subgroup analysis

#### HCC vs ICC in ≥ 50 years old

The IBR of HCC in patients ≥ 50 years old increased from 1978 to 2009 by 3.81%/year (p < 0.001), followed by a significant decrease between 2009–2018 at a rate of -2.54%/year (p < 0.001). In the same age group, the IBR of ICC increased by 9.98%/year (p < 0.001) from 1978 to 1997, followed by an insignificant decline (APC, -8.94%/year; p = 0.099) between 1997–2003, and then increased by 9.96%/year (p < 0.001) between 2003 and 2018.

Regarding IBMR of HCC in patients ≥ 50 years old increased by 2.98%/year (p < 0.001) from 1978 to 2010, followed by a significant decrease from 2010 to 2018 by -2.05%/year (p < 0.001). In the same age group, the IBMR of ICC rapidly increased by 9.88%/year (p < 0.001) from 1978 to 1997, followed by a significant decrease by -5.49%/year (p = 0.049) from 1997 to 2005, and then a rapid rise by 10.13%/year (p < 0.001) from 2005 to 2018.

#### Distant group

In the distant subgroup, the PLC IBR for individuals < 50 years old increased by 3.19%/year (p < 0.001) from 1978 to 1998, followed by a significant decline of -1.34%/year (p = 0.034) until 2018. In contrast, those ≥ 50 years old experienced a consistent increase in PLC IBR of 2.19%/year (p < 0.001) from 1978 to 2018.

For males, the distant PLC IBR rose by 1.72%/year (p < 0.001) throughout the study period. On the other hand, females had an initial increase of 1.53%/year (p < 0.001) from 1978 to 2010, followed by a sharper rise of 5.76%/year (p < 0.001) until 2018.

Regarding race, white individuals showed an increasing distant PLC IBR, first by 2.00%/year (p < 0.001) from 1978 to 2010, then accelerating to 4.03%/year (p = 0.001) from 2010 to 2018. Black individuals experienced a steady increase of 1.43%/year (p < 0.001) throughout the study period. In contrast, other races saw a decrease in distant PLC IBR by -0.82%/year (p = 0.001) throughout the study period.

In the distant subgroup, the PLC IBMR for individuals < 50 years old showed a significant decrease of -3.30%/year (p = 0.001) from 1978 to 1997, followed by a decrease of -1.71% per year (p = 0.005) from 1997 to 2018. For those aged ≥ 50, the distant PLC IBMR increased by 2.11%/year (p < 0.001) since 1978.

Regarding distal PLC IBMR in males and females, males had a persistent increase of 1.62%/year (p < 0.001) throughout the study period. Females initially saw an increase of 1.43%/year (p < 0.001) from 1978 until 2011, which then accelerated to 7.03%/year (p = 0.001) until 2018.

In terms of racial differences, the distant PLC IBMR for white individuals was stable between 1978 and 1991 (APC, 0.29%/year; p = 0.708), increased by 7.09% per year (p = 0.016) from 1991 to 1997, declined insignificantly by -2.37%/year (p = 0.338) from 1997 to 2003, and then increased again by 3.63%/year (p < 0.001) from 2003 onwards. For black individuals, there was a persistent increase in distant PLC IBMR by 1.37%/year (p < 0.001) throughout the study period. Other races’ rates declined by -0.85%/year (p = 0.001) over the entire study period.

## Discussion

### Incidence-based rates

Overall, PLC IBRs have steadily risen over the last four decades; however, since the mid-2000s, rates have plateaued. Since HCC accounts for the majority of PLC cases [4], better control of HCC risk factors likely explains this drop. Hepatitis C virus (HCV) is the most common cause of HCC in the US [15]. Advances in the pre-transfusion screening of blood products, HCV awareness, and treatment have been noted over the last 2 decades [16–18]. Furthermore, HCV direct-acting anti-viral therapy was approved in the US in 2011, which could also be contributing to declining incidence [19]. Although non-alcoholic fatty liver disease (NAFLD) incidences have been on the rise in the US [20, 21], it is unclear how this has affected HCC IBRs. Notably, recent studies suggested that patients with NAFLD, particularly the non-alcoholic steatohepatitis (NASH) subgroup, are more likely to die of cirrhosis, cardiovascular disease, diabetes mellitus, and non-HCC cancers than of HCC [22, 23]. Hence, the decrease in the incidences of PLC might be partially due to the rise of NAFLD. Further studies are needed to explore this correlation and whether PLC IBRs will continue to decline in the future.

PLC rates in individuals younger than fifty have been declining since the early 2000s. This can be partially explained by the introduction of Hepatitis B virus (HBV) vaccination and improved HBV treatment [24]. A randomized control trial showed that treatment of chronic HBV significantly decreases the risk of HCC development [25]. Those two factors might also explain the significant decline in PLC rates among Asians, who suffer from HBV the most [24]. On the other hand, the lack of a significant decline in IBRs in those older than fifty years could be explained by multiple factors. Firstly, with improved cirrhosis management, survival has improved; consequently, the risk of HCC among this group might be increasing. Moreover, a European study showed that a large proportion of patients with chronic HCV are late presenters and often present at an advanced age with complications such as HCC [26]. Finally, ICC is associated with advanced age, and patients with chronic HCV are at an increased risk of ICC [27] all of which might be contributing to the stabilization of the PLC rate among this age group in the setting of the overall decline in the study cohort. This is demonstrated in the subgroup analysis which showed that while HCC rates have started to decline, ICC rates have significantly increased, rising at more than three times the rate of the HCC decline. Although this increase in ICC rates might contribute to the overall rate stabilization in individuals older than 50 years of age, it is unlikely to be the sole factor.

Although it might be premature to conclude that a long-term decline in PLC has begun, the sensitivity analysis based on delay-adjusted rates offers assurance that the decrease in 2018 is not an artifact.

### Incidence-based mortality rates

The overall trend of PLC IBM increased till the early 2010s and then plateaued. The initial rise is concurrent with the obesity epidemic, which has spread steadily since the late 1990s [28, 29]. It is noteworthy to mention that obesity is prevalent in around forty percent of the US population above twenty years of age [30] and that obesity in itself is an independent risk factor of HCC mortality [31].

While males continue to have higher IBMRs than females, this study shows that the mortality gap between the two has been shrinking. This difference in IBMR between males and females could be related to the recent narrowing in the trends of alcohol consumption [32, 33]. In recent years, females have been consuming more alcohol [32–35]. Numerous studies have shown that females are more vulnerable to complications of alcohol consumption compared to males [33, 34, 36]. Around 2010, IBMRs in the white population plateaued while those in the black population have been declining. Multiple US studies showed that the prevalence of NAFLD and NASH among Whites was about 1.3 to 1.5 times more than Blacks [37, 38]. These factors could also be contributing to the observed disparity between the IBMRs in both races.

Regarding the subgroup analysis of HCC and ICC IBMR in patients ≥ 50 years old, the initial rise in ICC mortality parallels the increase in incidence and might be related to increased recognition of the disease [39]. The subsequent decrease in ICC mortality could be attributed to earlier detection and increased utilization of surgery, as a study has shown decreased mortality during a similar time frame [39]. However, the IBMR rose again significantly in this age group, which another study has attributed to increased surgical mortality in advanced age [40]. Additionally, another study found that while HCC incidence is decreasing, overall liver cancer mortality is increasing [41]. The former study cited this as evidence that the overall increase in PLC mortality, despite the decrease in HCC mortality, might be explained by the increase in ICC mortality, as our study also speculates [40].

Interestingly, there was an observed significant decline in IBMR amongst those less than fifty years old since 2000. In a similar timeframe, other races/ethnicities, including Asians, also showed a significant decline in IBMR. This could be due to the survival advantages of initiating anti-viral treatment in HCC patients with HBV/HCV; which are leading etiologies among these groups [42–44]. The introduction of screening and surveillance guidelines in the early 2010s also led to improved survival, decreased mortality, and the discovery of HCC at an earlier stage [45, 46]. However, our study also showed that despite an increase in the total PLC IBR, the IBR of localized PLC decreased from 2015–2018. This might seem contradictory, as screening typically leads to an increase in localized cases and a decrease in distal ones. However, the reported decrease in localized IBR was not statistically significant, possibly due to the limited three-year data. As such, unless future data confirms a potential decline, we assume that we are still in the plateau phase.

It is believed that the initiation of screening programs significantly contributed to the initial rise in localized PLC cases, which is supported by a study that showed that screening greatly increased the number of early HCC cases [47]. The subsequent plateau in localized PLC cases between 2015 and 2018 could be due to improved treatment of risk factors, as a recent study in the United Kingdom reported a similar plateau and attributed it to better treatment of hepatitis C [47]. However, as noted in the limitations section, access to data on risk factors was not available to further explain this trend.

### Limitations

As with all studies that use SEER as a data source, this study is affected by similar limitations. Firstly, the SEER registries do not provide information about etiologic factors, risk factors, or the presence of comorbidities; hence the proposed explanations for changes in PLC trends are speculative. Secondly, the results are dependent on the histological coding, whereby cases might be missing from the analysis due to variability in the coding. Finally, this paper shares the limitations of any retrospective study. Discussion on the limitations of SEER studies is beyond the scope of this paper; further details have been highlighted in a different report [48].

## Conclusion

The incidence and mortality of PLC have been increasing for decades, followed by a plateau in recent years. There seems to be a promising decline in mortality in some age (< 50) and race (Blacks/other races) sub-groups, but more effort is still needed to target modifiable risk factors for PLC that continue to burden a significant portion of the population. This should go hand in hand with the continued development of new diagnostic and therapeutic options.

## Abbreviations

APC: Annual percent change
HBV: Hepatitis B virus
HCC: Hepatocellular carcinoma
HCV: Hepatitis C virus
IBMR: Incidence-based mortality rates
IBR: Incidence-based rates
ICC: Intrahepatic cholangiocarcinoma
NAFLD: Nonalcoholic fatty liver disease
NASH: Nonalcoholic steatohepatitis
PLC: Primary liver cancer
US: United States
